# Five-year outcome of conventional and drug-eluting transcatheter arterial chemoembolization in patients with hepatocellular carcinoma

**DOI:** 10.1186/s12876-018-0848-1

**Published:** 2018-08-03

**Authors:** Yi-Sheng Liu, Chia-Ying Lin, Ming-Tsung Chuang, Chia-Ying Lin, Yi-Shan Tsai, Chien-Kuo Wang, Ming-Ching Ou

**Affiliations:** Department of Diagnostic Radiology, National Cheng Kung University Hospital, College of Medicine, National Cheng Kung University, No. 138 Sheng Li Road, Tainan, 704 Taiwan, Republic of China

**Keywords:** Drug-eluting bead transcatheter arterial chemoembolization, DEB-TACE, Hepatocellular carcinoma, Transcatheter arterial chemoembolization, TACE

## Abstract

**Background:**

Currently, no standard of care or therapies have been established for patients with advanced HCC. We evaluated the efficacy and safety of conventional transarterial chemoembolization using gelatin sponges or microspheres plus lipiodol-doxorubicin (cTACE) and TACE with doxorubicin-loaded drug eluting beads (DEB-TACE).

**Methods:**

This retrospective study included 273 patients who received cTACE (*n* = 201) or DEB-TACE. Tumor response, survival, and adverse events were evaluated over a 5-year follow-up period.

**Results:**

During 5-year follow-up, a greater percentage of patients treated with cTACE died than those treated with DEB-TACE (76.1% vs. 66.7%) (*P* = 0.045). At the last evaluation, all surviving patients had disease progression and no differences were seen between treatment groups. However, the time to disease progression differed between groups; median time to disease progression was 11.0 months for cTACE and 16.0 months for DEB-TACE (*P* = 0.019). The median survival time was 37 months in both treatment groups. No significant differences were observed between cTACE and DEB-TACE therapies in subgroups of patients with BCLC stage A or stage B + C either in survival time or time to disease progression (*P* values > 0.05). No significant differences were observed in survival status or disease progression between cTACE and DEB-TACE in patient subgroups with either tumor number > 5 or with the sum of the diameter of largest five HCC tumors being > 7 cm.

**Conclusions:**

DEB-TACE demonstrates greater long-term benefits than cTACE in treating treatment-naïve patients with HCC. Results of this long-term study support the use of DEB-TACE in treating HCC.

## Background

Hepatocellular carcinoma (HCC) is the third leading cause of cancer-related deaths worldwide and the sixth most common cancer [1, 2]. Due to chronic liver infection resulting from the high incidence of hepatitis C infection and the large number of persons with metabolic syndrome, the incidence of HCC is anticipated to rise [1, 2]. A large percentage of HCC patients are diagnosed at the intermediate or advanced stage [3, 4]. Currently, no established standard of care or therapeutic possibilities exist for patients with advanced HCC. Only 30 to 40% patients with HCC are candidates for curative treatment such as liver transplant [3, 4]. Hence, most patients can only be treated with locoregional or palliative treatment [5].

Transcatheter arterial chemoembolization (TACE) is used as a palliative local therapeutic option for patients with nonresectable HCC who may be waiting for liver transplant or who are not candidates for liver transplantation. TACE is also used to decrease the tumor burden, allowing for tumor resection [6]. Conventional TACE (cTACE) involves the delivery of embolic material to the tumor plus chemotherapeutic agents such as doxorubicin, either dissolved or emulsified in lipiodol, which is noted for causing both ischemia and strong cytotoxic effects [2, 7]. Lipiodol is a lymphographic agent which is selectively deposited in HCC tumors by arterial infusion and permits the slow release of the chemotherapeutic agent into the tumor [8–10]. In randomized controlled trials, cTACE has demonstrated a survival benefit for HCC patients with nonresectable HCC [11–14] and is recommended as a standard treatment option for patients with Barcelona stage B (BCLC) (intermediate stage) HCC [15, 16]. However, with TACE, the tumor does not always retain the lipiodol, resulting in decreased effectiveness of therapy and risk of liver damage [17, 18].

Drug-eluting beads (DEB) have been introduced into TACE (DEB-TACE) to promote the controlled release of cytotoxic drugs for the treatment of HCC [19, 20]. The drug-eluting microspheres added to TACE result in the delivery of high concentration of chemotherapeutic drugs to the tumor. The use of DEB-TACE is associated with a better safety profile than cTACE; DEB-TACE has been observed to have a lower incidence of adverse events such as abdominal pain, fever, nausea and vomiting [21–24]. A number of clinical studies and meta-analyses have found that DEB-TACE was associated with a significant advantage in tumor response and survival compared with cTACE [21–24]. However, several recent studies failed to demonstrate the superiority of DEB-TACE over cTACE in treatment efficacy, although DEB-TACE was associated with a better safety profile than cTACE [25–28].

Although DEB-TACE has shown benefits relative to TACE in some randomized controlled studies, the method is still controversial in clinical practice. Early results of our study of DEB-TACE and cTACE published in 2015 [29] showed that, at a mean follow-up of 15 months, DEB-TACE was associated with a better safety profile and more patients achieved a complete response and fewer had disease progression than cTACE. The present retrospective study evaluated the long-term benefits of DEB-TACE and cTACE on disease progression and overall survival (OS) during 5-year follow up of patients with HCC.

## Methods

This retrospective study recruited consecutive patients with HCC who were treated with TACE at the National Cheng-Kung University Hospital (Tainan, Taiwan) from November 2010 to November 2011. The retrospective analysis was approved by the Ethics Committee of the institutional review board, National Cheng Kung University Hospital (IRB No: A-ER-103-311). The study was performed in accordance with the Declaration of Helsinki and the protocol was reviewed and approved by the Institutional Review Board of the hospital. All patients provided signed informed consent.

### Study patients

The study design and protocol were as described previously in Liu et al. [29]. Eligible patients were ≥ 18 years of age with a diagnosis of HCC, had a at least one tumor that had not been treated previously and was > 1 mm in diameter, had Barcelona Clinic Liver Cancer (BCLC) criteria A or B, and had an Eastern Cooperative Oncology Group (ECOG) performance score of 0 or 1. Included patients were also required to have a serum creatinine of < 1.2 mg/dL, aspartate aminotransferase (AST) and alanine aminotransferase (ALT) levels < 100 IU/L, and total bilirubin < 3 mg/dL. Patients were excluded if the tumor had invaded the portal vein, hepatic vein, and/or biliary duct, if the tumor had an extrahepatic arterial supply, or if the patient was diagnosed with atypical HCC such as, for example, infiltrative.

### Treatment

A multidisciplinary team determined the treatment for a given patient. All patients were treated with a single cycle of TACE. Study patients either received cTACE with a gelatin sponge or with Embosphere microspheres (Biosphere, Roissy, France), or chemoembolization with doxorubicin-containing DEB (DC Bead, Biocompatibles, Farnham, United Kingdom). Prior to treatment, the attending physician described to the patient the tumor response and complication rates for each treatment method, as determined by the published literature. Subsequently, the patient decided which method should be used. No other simultaneous or combined treatment was permitted during the cTACE or DEB-TACE treatment period.

On the day of treatment, the patient underwent a complete diagnostic angiographic evaluation of the hepatic artery, superior mesenteric artery, and celiac trunk so as to evaluate the vascular anatomy and portal flow [30]. The segmental and subsegmental arteries feeding the tumor were subsequently catheterized using super-selective angiography with a microcatheter. The right hepatic artery was used in patients whose right hepatic artery came from the superior mesenteric artery. Care was taken to avoid embolization of the cystic and falciform arteries. The phrenic artery was investigated if it was determined to be supplying the tumor.

Patients in the cTACE group were injected with the doxorubicin/lipiodol mixture consisting of 50 mg of doxorubicin mixed with 10 mL of lipiodol. The mixture was injected into a segmental or subsegmental artery, followed by an injection of 500 to 700 μm gelatin sponge (Spongostan standard, Johnson & Johnson, Gargrave, Skipton, United Kingdom), or 100 to 300 μm Embosphere microspheres. The amount of lipiodol/doxorubicin injected was determined by the tumor size [31]. The DEB-TACE group was injected with 2 mL of 300 to500 μm DEB combined with 70 mg of doxorubicin [32]. An additional volume(s) of DEB was injected if “near stasis” was not obtained after the first injection until “near stasis” was achieved. The amount of beads injected was based upon the manufacturer’s instructions. The study used 300 to 500 μm beads because the 100 to 300 μm beads were not yet approved in Taiwan at the time the study started.

### Follow-up and evaluation of treatment response

Tumor status was evaluated every 3 to 4 months according to the modified Response Evaluation Criteria in Solid Tumors (mRECIST) [33]. If the evaluation suggested partial response or stable disease, the patients continued to be followed every 3 to 4 months for up to and over 5 years. Partial response was defined as ≥30% reduction in the sum of the diameter of the visible target lesions compared with baseline. If the assessment indicated progressive disease, patients were treated according to the BCLC guidelines and disease status [34]. Progressive disease was defined using the mRECIST criteria; i.e., ≥20% increase in the sum of the diameters of the visible target lesions compared with the smallest measurements observed from the start of therapy. Stable disease was defined as cases in which the tumor evaluation did not meet the criteria of partial response or progressive disease [33]. Two experienced radiologists evaluated the images, and any discrepancies were resolved by consensus.

Safety was evaluated throughout the study.

### Statistical analysis

Clinical data are summarized as mean ± standard deviation (SD) and n (%) for continuous and categorical variables by treatment. Differences between treatments were compared using Mann-Whitney U test for continuous variables and Chi-square test / or Fisher’s exact test for categorical variables and for survival time and progression time. Pearson Chi-square test / or Fisher’s exact test were used to evaluate survival and progression status between treatments. The overall survival time and progression time by treatment were graphed using Kaplan-Meier curve and the estimated survival time was presented as median with 95% confidence intervals (95% CI). The log-rank test was applied to compare the difference in OS time between treatments. Results are represented as a *P* value. All statistical assessments were two-tailed and considered significant at *P* < 0.05. All statistical analyses were carried out using IBM SPSS statistical software version 22 for Windows (IBM Corp. Released 2013. IBM SPSS Statistics for Windows, Version 22.0. Armonk, NY.).

## Results

### Baseline demographics and disease characteristics

A total of 273 patients (187 males / 86 females) with a mean age of 64.4 years were included in the study (Table 1). Of these, 201 patients were treated with cTACE and 72 with DEB-TACE. The baseline clinical characteristics were similar between treatment groups except for age, CLIP stage, and BCLC stage; patients in the cTACE group were older (65.3 vs. 61.7 years of age; *P* = 0.009), a greater percentage of patients had CLIP stage 0 or 1 cancer (81.6% vs. 66.7%; *P* = 0.018)) and BCLC A stage disease (35.7% vs. 22.2%; *P* = 0.040) than patients in the DEB-TACE group. Across groups, 63% of patients had no prior treatment, about 50% had unilobar disease, and the majority had HCV or HBV infections. About 80% of patients had Child-Pugh stage 5 disease and approximately 99% had an ECOG status of 0. The mean largest tumor size was 3.64 cm and most patients (72.2%) had ≤5 tumors.

**Table 1:** Patients’ clinical characteristics by cTACE and DEB-TACE groups. (*N* = 273)

Variables	Total (*N* = 273)	cTACE (*n* = 201)	DEB-TACE (*n* = 72)	*p*-value
Age, years	64.4 ± 10.7	65.3 ± 10.7	61.7 ± 10.3	**0.009**
Sex				
Female	86 (31.5)	63 (31.3)	23 (31.9)	0.925
Male	187 (68.5)	138 (68.7)	49 (68.1)	
Previous treatment				
None	172 (63.0)	126 (62.7)	46 (63.9)	0.166
Operation	53 (19.4)	35 (17.4)	18 (25.0)	
Locoregional treatment	41 (15.0)	33 (16.4)	8 (11.1)	
Operation + locoregional	7 (2.6)	7 (3.5)	0 (0)	
Uni-/Bi-lobar				
Unilobar	136 (49.8)	106 (52.7)	30 (41.7)	0.107
Bilobar	137 (50.2)	95 (47.3)	42 (58.3)	
HBV/HCV				
Non-B or C	22 (8.1)	18 (9.0)	4 (5.6)	0.225
HBV	127 (46.5)	87 (43.3)	40 (55.6)	
HCV	108 (39.6)	82 (40.8)	26 (36.1)	
HBV + HCV	16 (5.9)	14 (7.0)	2 (2.8)	
GOT (IU/L)	70.63 ± 48.72	69.88 ± 50.4	70.63 ± 48.72	0.331
GOT				
Not normal	156 (57.1)	112 (55.7)	44 (61.1)	0.428
Normal	117 (42.9)	89 (44.3)	28 (38.9)	
GPT (IU/L)	72.08 ± 80.27	69.97 ± 81.77	72.08 ± 80.27	0.109
GPT				
Not normal	132 (48.4)	91 (45.3)	41 (56.9)	0.089
Normal	141 (51.6)	110 (54.7)	31 (43.1)	
Bilirubin (mg/dL)	0.76 ± 0.44	0.73 ± 0.42	0.76 ± 0.44	0.087
Bilirubin				
Not normal	22 (8.1)	13 (6.5)	9 (12.5)	0.107
Normal	251 (91.9)	188 (93.5)	63 (87.5)	
AFP (ng/mL)	875.28 ± 4510.43	766.21 ± 4607.3	875.28 ± 4510.43	0.497
AFP				
Negative	224 (82.1)	169 (84.1)	55 (76.4)	0.145
Positive	49 (17.9)	32 (15.9)	17 (23.6)	
Albumin (g/dL)	4 ± 0.49	3.98 ± 0.48	4 ± 0.49	0.312
Albumin				
Not normal	41 (15.0)	29 (14.4)	12 (16.7)	0.648
Normal	232 (85.0)	172 (85.6)	60 (83.3)	
Ascites				
None	257 (94.1)	191 (95.0)	66 (91.7)	0.432
Mild	15 (5.5)	9 (4.5)	6 (8.3)	
Moderate	1 (0.4)	1 (0.5)	0 (0)	
Child-Pugh stage				
5	219 (80.2)	161 (80.1)	58 (80.6)	0.883
6	44 (16.1)	33 (16.4)	11 (15.3)	
7	9 (3.3)	6 (3.0)	3 (4.1)	
8	1 (0.4)	1 (0.5)	0 (0)	
ECOG stage				
0	271 (99.3)	199 (99.0)	72 (100)	1.000
1	2 (0.7)	2 (1.0)	0 (0)	
CLIP stage				
0	40 (14.7)	32 (15.9)	8 (11.1)	**0.018**
1	172 (63.0)	132 (65.7)	40 (55.6)	
2	49 (17.9)	29 (14.4)	20 (27.8)	
3	10 (3.7)	8 (4.0)	2 (2.8)	
4	2 (0.7)	0 (0)	2 (2.8)	
Okuda stage				
0	250 (91.6)	185 (92.0)	65 (90.3)	0.471
1	20 (7.3)	13 (6.5)	7 (9.7)	
2	3 (1.1)	3 (1.5)	0 (0)	
BCLC stage				
A	87 (32)	71 (35.7)	16 (22.2)	**0.040**
B + C	184 (67.9)	128 (64.3)	56 (77.8)	
Largest target (cm)	3.64 ± 2.59	3.47 ± 2.32	4.12 ± 3.20	0.175
Tumor numbers	3.5 ± 1.9	3.4 ± 1.9	3.6 ± 2.0	0.690
Tumor numbers ≤5	197 (72.2)	148 (73.6)	49 (68.1)	0.365
Tumor numbers > 5	76 (27.8)	53 (26.4)	23 (31.9)	
Sum of the largest five hepatocellular carcinoma diameter (cm)	6.64 ± 2.33	6.63 ± 2.26	6.66 ± 2.53	0.908

Aspartate aminotransferase (GOT), Alanine aminotransferase (GPT)

Data are summarized as mean ± SD and n (%) for continuous and categorical variables by treatment. Differences between treatments were compared using Mann-Whitney U test for continuous variables and Chi-square test / or Fisher’s exact test for categorical variables

Bold *p*-values indicate statistical significance (*p* < 0.05)

### Treatment response

Clinical characteristics after treatment differed between cTACE and DEB-TACE patients (Table 2). Mean aspartate aminotransferase (GOT), alanine aminotransferase (GPT) and bilirubin were significantly different between the two groups. DEB-TACE patients had lower mean GOT (73.31 ± 45.50), and higher percentages had normal GOT values (36.1%). The same was also true for bilirubin levels; DEB-TACE patients had lower mean (0.98 ± 0.57) and higher percentages with normal bilirubin values (80.6%). Compared to cTACE patients, DEB-TACE patients also had significantly lower mean GPT (94.17 ± 101.44), but the percentage of patients with normal values was similar between the two groups. No significant differences were found in the percentage of patients with cholangitis between the cTACE and DEB-TACE groups. (Table 2).

**Table 2:** Patients’ clinical characteristics after treatment with cTACE and DEB-TACE. (*N* = 273)

Variables	cTACE (*n* = 201)	DEB-TACE (*n* = 72)	*p*-value
GOT (IU/L)	156.76 ± 176.58	73.31 ± 45.50	**< 0.001**
GOT			
Not normal	153 (76.1)	46 (63.9)	**0.045**
Normal	48 (23.9)	26 (36.1)	
GPT (IU/L)	170.91 ± 266.13	94.17 ± 101.44	**0.014**
GPT			
Not normal	139 (69.2)	47 (65.3)	0.545
Normal	62 (30.8)	25 (34.7)	
Bilirubin (mg/dL)	1.38 ± 0.99	0.98 ± 0.57	**0.002**
Bilirubin			
Not normal	75 (37.3)	14 (19.4)	**0.006**
Normal	126 (62.7)	58 (80.6)	
Albumin (g/dL)	3.81 ± 0.43	3.87 ± 0.42	0.325
Albumin			
Not normal	37 (18.4)	12 (16.7)	0.741
Normal	164 (81.6)	60 (83.3)	
Cholangitis			
No	193 (96)	70 (97.2)	0.641
Yes	8 (4)	2 (2.8)	

Aspartate aminotransferase (GOT), Alanine aminotransferase (GPT)

Data are summarized as mean ± SD and n (%) for continuous and categorical variables by treatment. Differences between treatments were compared using Mann-Whitney U test for continuous variables and Chi-square test / or Fisher’s exact test for categorical variables

All serious AE were classified as cholangitis, none as biloma

Bold *p*-values indicate statistical significance (*p* < 0.05)

**Fig. 1:**
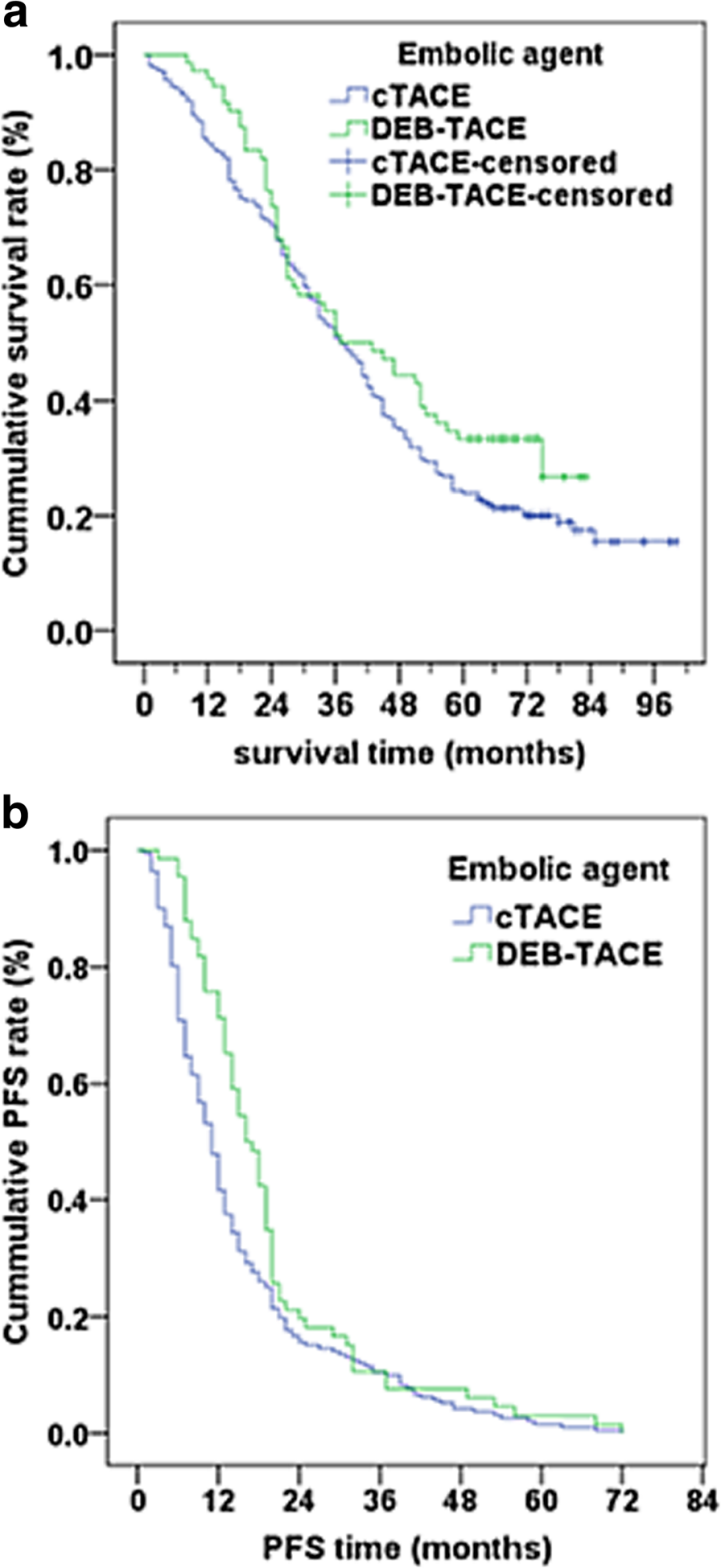
Kaplan-Meier curve of overall survival time (**a**) and PFS time (**b**) by treatments. **a** The estimated median overall survival time was derived as 37 months (95%CI = 32.2–41.8 months) for cTACE and 37 months (95%CI = 23.5–50.5 months) for DEB-TACE. The log-rank test p-value = 0.091. **b** The estimated median PFS time was derived as 11 months (95%CI = 9.6–12.4 months) for cTACE and 16 months (95%CI = 13.1–18.9 months) for DEB-TACE. The log-rank test *p*-value = 0.019

Survival time for the overall population was median 37 months (95% CI = 32.2–41.8 months) for cTACE treatment and a similar median 37 months (95%CI = 23.5–50.5 months) for the DEB-TACE treatment group (*P* = 0.091) (Table 3 and Fig. 1a). Over the 5-year follow-up period, a greater percentage of patients treated with cTACE died than those treated with DEB-TACE (76.1% vs. 66.7%, respectively) (*P* = 0.045). All patients who were still alive at the last evaluation (15 patients died during the 5-year follow-up) had disease progression (Table 3). However, the time to disease progression differed between groups; median time to disease progression was 11.0 months for cTACE and 16.0 months for DEB-TACE (*P* = 0.019). (Table 3 and Fig. 1b).

**Table 3:** Survival times and progression times between cTACE and DEB-TACE treatments

Variables	cTACE	DEB-TACE	*p*-value
Total	(*n* = 201)	(*n* = 72)	
Survival status			**0.045**
Died within ≤5 years follow-up	153 (76.1)	48 (66.7)	
Died after more than 5 years follow-up	10 (5.0)	1 (1.4)	
Survived until last follow-up	38 (18.9)	23 (31.9)	
Progression status			0.218
Progression	192 (95.5)	66 (91.7)	
Loss of follow-up/censored	9 (4.5)	6 (8.3)	
Survival time, months	37 (32.2, 41.8)	37 (23.5, 50.5)	0.091
Progression time, months	11.0 (9.6, 12.4)	16.0 (13.1, 18.9)	**0.019**
BCLC stage = A	(*n* = 73)	(*n* = 16)	
Survival status			0.083
Died within ≤5 years follow-up	57 (78.1)	9 (56.3)	
Died after more than 5 years follow-up	3 (4.1)	0 (0)	
Survived until last follow-up	13 (17.8)	7 (43.8)	
Progression status			0.219
Progression	70 (95.9)	14 (87.5)	
Loss of follow-up/censored	3 (4.1)	2 (12.5)	
Survival time, months	42 (36.1, 47.9)	45 (0, 90.1)	0.149
Progression time, months	12 (10.5, 13.5)	19 (17.8, 20.2)	0.217
BCLC stage = B + C	(*n* = 128)	(*n* = 56)	
Survival status			0.270
Died within ≤5 years follow-up	96 (75)	39 (69.6)	
Died after more than 5 years follow-up	7 (5.5)	1 (1.8)	
Survived until last follow-up	25 (19.5)	16 (28.6)	
Progression status			0.495
Progression	122 (95.3)	52 (92.9)	
Loss of follow-up/censored	6 (4.7)	4 (7.1)	
Survival time, months	33 (27.1, 38.9)	36 (21.3, 50.7)	0.191
Progression time, months	10 (8.1, 11.9)	15 (12.4, 17.7)	**0.032**

Survival and progression status are summarized as n (%) by treatment; Survival time- and progression time-related data are summarized as median (95%CI) by treatment

Differences between treatments were compared using Pearson Chi-square test / or Fisher’s exact test for survival and progression status and Log-rank test for survival time or progression time

Bold *p*-values indicate statistical significance (*p* < 0.05)

Survival time and time to disease progression between treatments was also evaluated by BCLC stage, and by patients with > 5 tumors or whose sum of the five largest tumors was > 7 cm in diameter. No significant differences were observed between cTACE and DEB-TACE therapies in the subgroups of patients with BCLC stage A or stage B + C, either in survival time or progression time (*P* values > 0.05) (Table 3). In addition, no significant differences were observed in survival status or disease progression between cTACE and DEB-TACE in the subgroups of patients with either tumor number > 5 or the sum of the diameter of the five largest HCC tumors was > 7 cm. (Table 4).

**Table 4:** Survival times and progression times between cTACE and DEB-TACE treatments for patients with tumor number > 5 or sum of the diameter of the largest five HCC tumors was > 7 cm

Variables	cTACE	DEB-TACE	*p*-value
	(*n* = 78)	(*n* = 33)	
Survival status			0.610
Died within ≤5 years follow-up	62 (79.5)	26 (78.8)	
Died after more than 5 years follow-up	3 (3.8)	0 (0)	
Survived until last follow-up	13 (16.7)	7 (21.2)	
Progression status			0.360
Progression	75 (96.2)	30 (90.9)	
Loss of follow-up/censored	3 (3.8)	3 (9.1)	
Survival time, months	31 (26.7, 35.3)	27 (22.3, 31.7)	0.789
Progression time, months	9 (6.7, 11.3)	12 (8.0, 16.0)	0.492

Survival and progression status are summarized as n (%) by treatment; Survival time- and progression time-related data are summarized as median (95%CI) by treatment

Differences between treatments were compared using Pearson Chi-square test / or Fisher’s exact test for survival and progression status and Log-rank test for survival time or progression time

## Discussion

This retrospective, observational study compared clinical outcomes of treatment-naive HCC patients who underwent cTACE (conventional TACE) or DEB-TACE (drug-eluting bead TACE). The earlier published findings from the 15-month follow-up of this study population showed that DEB-TACE treatment resulted in a higher percentage of patients with complete response and a lower percentage with disease progression than patients receiving cTACE treatment [29]. In addition, DEB-TACE was associated with a better safety profile than cTACE [29]. The data presented here are those of the same study after 5-years of follow-up. Five-years after treatment, both treatments were associated with a survival time of 37 months. However, over the 5-year follow-up period, a greater percentage of patients treated with cTACE died (76.1%) than those treated with DEB-TACE (66.7%) (*P* = 0.045). After five-years, all surviving patients had disease progression. However, the time to disease progression differed between groups; cTACE was associated with a shorter median time to disease progression (11 months) than DEB-TACE (16.0 months) (*P* = 0.019). Subgroup analysis indicated that survival and disease progression were similar for both treatments in patients with BLCC stage A or stage B + C, and in patients with > 5 tumors, or if the diameter of the patient’s five largest tumors was > 7 cm. The findings of the long-term study suggest that DEB-TACE demonstrates long-term benefits in treating treatment-naive HCC patients.

Only a limited number of studies have directly compared the efficacy and safety of DEB-TACE and cTACE in treating HCC. A number of systematic reviews and meta-analyses have evaluated the effectiveness of cTACE and DEB-TACE in treating HCC [21–24].

Similar to our findings, several of the meta-analyses found DEB-TACE to show treatment benefit and better safety profile compared with cTACE [23, 24]. A systematic review by Martin et al. [21] found DEB-TACE had a significant advantage compared with cTACE in objective response and had greater overall disease control in patients with advanced HCC (*P* values ≤0.038). Zhou et al. performed a meta-analysis which included nine studies and total of 830 patients [24]. The study found DEB-TACE significantly improved overall survival and increased objective response and disease control rates. Similarly, a meta-analysis performed by Huang et al. [23], which included seven clinical studies with 700 patients, found a significantly better objective response for DEB-TACE than cTACE (*P* = 0.004), with a relative risk difference of 0.15 (*P* = 0.0003). Those authors also found that patients with one- and two-year survival were significantly more with DEB-TACE than with cTACE (*P* values ≤0.007). However, Huang et al. found no difference between treatments for 6-month and 3-year survival (*P* values ≥0.11). A meta-analysis by Zou et al. [22] found DEB-TACE was associated with higher complete response (Odds ratio [OR], 1.35), overall survival rate (OR, 1.41), and survival time (WMD, 3.03). In contrast to the studies of Huang et al. and Zhou et al., Zou et al. found no difference between treatments for objective response. Those authors also found that the treatments had similar disease control rates. All four meta-analyses found that DEB-TACE was associated with fewer side effects compared with cTACE.

In contrast to the above results, a meta-analysis performed by Facciorusso et al. [25] found that DEB-TACE and cTACE had similar safety and effectiveness. Facciorusso et al. included 12 studies, four of which were randomized controlled trials. The meta-analysis included a total of 1449 patients. The observed 1-, 2- and 3-year survival times were similar between treatment groups (*P* values ≥0.06). Pooled data of objective response and incidence of adverse events also indicated no differences between the two therapies (P values ≥0.36). However, the study did show a non-significant trend of superiority for DEB-TACE for overall response rate (OR, 1.2), which was confirmed by subgroup and sensitivity analysis.

The difference in findings across the meta-analyses may, in part, reflect the small number of included studies and high heterogeneity in the studies overall. Moreover, the evaluation of response rate across the studies varied, with some of the included studies used EASL criteria and others using mRECIST criteria. In addition, length of follow-up was heterogeneous among the included studies and was found to be one of the main sources of heterogeneity. The present study supports the importance of follow-up in comparing studies. In an earlier phase of the present t study conducted at 15 months post-treatment, DEB-TACE treatment was associated with patients’ greater complete response and less disease progression than cTACE. However, at the 5-year follow-up, all surviving patients had disease progression but fewer patients treated with DEB-TACE had died.

The present study has several limitations, including that the study was retrospective in design and only included patients from a single institution. In addition, complete secondary treatment information was not available for the patients and only a small number of patients were treated with DEB-TACE (*n* = 73).

## Conclusions

In conclusion, DEB-TACE shows greater long-term benefit compared with those of cTACE in treating treatment naïve patients with HCC. At 5 years after treatment, HCC patients receiving DEB-TACE have fewer deaths and a longer time to disease progression than patients receiving cTACE. DEB-TACE is also associated with fewer treatment-related adverse events than cTACE. Results of this long-term study support the use of DEB-TACE in treating HCC.

## Abbreviations

cTACE: Conventional transarterial chemoembolization
DEB: With doxorubicin-loaded drug eluting beads
ECOG: Eastern cooperative oncology group
HCC: Hepatocellular carcinoma
mRECIST: Modified response evaluation criteria in solid tumors
OS: Overall survival
TACE: Transarterial chemoembolization
TACE: Transarterial chemoembolization
BCLC: Barcelona clinic liver cancer
